# Comparison of radiation regimens in the treatment of Glioblastoma multiforme: results from a single institution

**DOI:** 10.1186/s13014-015-0396-6

**Published:** 2015-04-26

**Authors:** Melissa Azoulay, Fabiano Santos, Luis Souhami, Valerie Panet-Raymond, Kevin Petrecca, Scott Owen, Marie-Christine Guiot, Mariia Patyka, Siham Sabri, George Shenouda, Bassam Abdulkarim

**Affiliations:** Department of Oncology, Division of Radiation Oncology, McGill University, Montreal General Hospital, 1650 Avenue Cedar, H3G 1A4 Montréal, QC Canada; Department of Oncology, Division of Cancer Epidemiology, McGill University, 546 Pine Avenue West, H2W1S6 Montreal, QC Canada; Department of Neurology and Neurosurgery, Montreal Neurological Hospital, McGill University, 3801 University Avenue, H2W1S6 Montreal, QC Canada; Division of Medical oncology, Department of Oncology, Montreal General Hospital, McGill University, 1650 Avenue Cedar, H3G 1A4 Montréal, QC Canada; Department of Pathology, Montreal Neurological Hospital, McGill University, 3801 University Avenue, H3A 2B4 Montreal, QC Canada; Research Institute of the McGill University Health Center, Montreal General Hospital, 1625 Pine Avenue West, H3G 1A4 Montreal, QC Canada

**Keywords:** Glioblastoma, Radiation, Hypofractionation, Temozolomide, MGMT

## Abstract

**Background:**

The optimal fractionation schedule of radiotherapy (RT) for Glioblastoma multiforme (GBM) is yet to be determined. We aim to compare different fractionation regimens and identify prognostic factors to better tailor RT for newly diagnosed GBM patients.

**Methods:**

All data for patients who underwent surgery for GBM between January 2005 and December 2012 were compiled. Clinical information was collected using patient charts and government registry. Cox analysis was used to identify variables affecting survival and treatment outcome.

**Results:**

The median follow-up time was 13.2 months. Two hundred and seventy-six patients met the inclusion criteria, including 147 patients in the 60 Gy in 30 fractions (ConvRT) group, 86 patients in the 60 Gy in 20 fractions (HF60) group, and 43 patients in the 40 Gy in 15 fractions (HF40) group. Median survival (MS) was 16.0 months with a median progression-free survival (PFS) of 9.23 months in the ConvRT group. This was comparable to outcome in the HF60 group with MS 15.0 months and a median PFS of 9.1 months. Patients in the HF40 group had MS of 8 months, with a median PFS 5.4 months. Cox analysis showed no significant difference in OS between the ConvRT and HF60 groups but worse outcome in the HF40 group (HR 2.22, *P* = 0.04). MGMT methylation, extent of resection, use of chemotherapy, and repeat surgery were found to be significant independent prognostic factors for survival.

**Conclusions:**

HF60 constitutes a safe RT approach that shows survival comparable to standard RT while allowing for a shorter treatment time.

## Introduction

Glioblastoma multiforme (GBM) is the most lethal form of primary brain tumor in adults. Maximal safe resection followed by radiotherapy (RT) with concomitant and adjuvant temozolomide (TMZ) is the current standard of care [1,2]. O6-methylguanine-DNA methyltransferase (MGMT) is a DNA-repair protein that protects GBM tumor cells against alkylating agents by removing alkyl adducts from the O6-position of guanine [3]. MGMT promoter methylation has been accepted as a significant prognostic biomarker, with a median survival of 23.4 months for GBM patients with methylated MGMT, compared to 12.6 months in the case of unmethylated tumors [4].

The current standard RT regimen for GBM involves the delivery of 60 Gy in 2.0 Gy per fraction, delivered over 6 weeks. Hypofractionation refers to the use of a fewer number of larger sized fractions to reduce the overall treatment time, limit tumor repopulation, and potentially increase cell kill [5,6]. At this time, hypofractionation has been administered mostly to patients over 65 years of age and/or with poor performance status, patients who might derive only limited benefit from combined chemoradiation [4,7,8]. However, previous retrospective studies have suggested that hypofractionated RT regimen could result in survival comparable to conventional RT [9-12]. In a retrospective study, we reported on 35 patients who were treated with a hypofractionated RT regimen while receiving concomitant and adjuvant TMZ [10]. The outcome was comparable to the EORTC/NCIC data with a median overall (OS) survival of 14.4 months and minimal toxicity.

A number of prospective trials have also reported on the safety and efficacy of the hypofractionated approach [13-16]. However, concerns over the potential toxicity associated with a high dose, such as radionecrosis, have hindered the adoption of hypofractionated RT. A prospective study in the pre-TMZ era reported on the delivery of 50 Gy in 5 Gy/fraction daily to the enhancing primary disease, residual tumor, and surgical cavity [13]. The outcome was comparable to the survival data at that time, with a median OS of 7 months after treatment completion. The development of intensity modulated radiation therapy (IMRT) has allowed for more conformal RT treatment with better dose modulation, thereby decreasing the risk of complications. Our group reported a phase I trial that included 25 newly diagnosed GBM patients treated with hypofractionated IMRT and TMZ [14]. The median survival was 15.67 months. More recently, a phase II trial by Luchi et al. reported on their use of a concomitant radiation boost of 68 Gy in 8 fractions encompassing the surgical cavity and residual [15]. They did not report any neurological toxicity and the median OS was 20.0 months.

The optimal fractionated RT regimen in GBM remains to be determined, with the objective to improve local control and to reduce toxicity. Despite a number of studies supporting the use of hypofractionated RT, the current conventional RT regimen of 60 Gy in 2 Gy per fraction is still the standard of care in GBM. Our study investigates the benefits of hypofractionation in a population-based cohort of patients diagnosed with GBM.

## Materials and methods

### Patient population

After obtaining GEN-Research Ethics Board ethics board approval, we compiled a list of all adult patients who underwent surgery for histologically-confirmed GBM between January 2005 and December 2012. Patient information, tumor characteristics including MGMT status, treatment details, and outcomes were obtained using computerized hospital charts and patient registry. Of the 475 patients identified, a total of 342 patients received adjuvant RT, with or without concomitant TMZ. From this group, 66 patients were excluded from the final analysis for not having completed a curative RT treatment to a dose of at least 40 Gy, having a history of prior cranial RT, and/or having infra-tentorial GBM lesions.

#### Patient management

All patient cases were reviewed by a multidisciplinary group and offered surgery, adjuvant RT and/or chemotherapy. All tumors diagnosed as GBM were reviewed by the neuropathologists.

#### Surgery

Maximum safe resection was performed by the neurosurgeon and was based on tumor location, extent of disease, and on patient characteristics, which included KPS, age, and co-morbidities. Presence of residual disease was assessed via gadolinium-enhanced MRI performed at 48-72 hours post-operatively in close to 90% of patients. Operative reports were used in cases where imaging was not available.

### Radiation treatment regimen

Patients were immobilized with a thermoplastic mask for simulation and treatment. They underwent CT-based planning with 3 mm slices. Co-registration of the post-operative MRI was performed, when imaging was available. Enhancing residual disease, if present, as well as the surgical cavity were contoured. A margin of 2 cm was added with appropriate cropping to respect anatomical boundaries. For patients who received the hypofractionated regimen, the treatment planning included two planning target volumes (PTV): (i) PTV40 was defined by adding a 1.5 cm margin on the GTV/cavity and it received 40 Gy in 20 fractions; (ii) PTV60 was a concomitant boost volume defined by adding a 0.5 cm margin on the GTV/residual and it received a total dose 60 Gy in 20 fractions. Radiation treatment was planned using either forward or inverse IMRT. In most cases, the PTV was covered by the 95% isodose. Organs at risk including the brainstem, optic chiasm, and optic nerves were contoured. Dose to the brainstem and optic chiasm was limited to less than 54 Gy for conventional fractionation. Dose constraints for the hypofractionated regimen were specified as less than 40 Gy in 20 fractions to the brainstem, chiasm, and at least one retina.

The following fractionation schedules were used: conventional RT with 60 Gy in 30 fractions (ConvRT); hypofractionated RT with 60 Gy in 20 fractions (HF60); hypofractionated RT with 40 Gy in 15 fractions (HF40). All the regimens were delivered 5 days per week. The HF60 regimen excluded patients with T1 gadolinium enhancement present at less than 1.5 cm from the brainstem and/or optic apparatus. Furthermore, patients over the age of 70 or with KPS <70 usually received the HF40 regimen, as per current treatment guidelines [17].

### Chemotherapy

The majority of patients received concomitant TMZ at a dose of 75 mg/m2 daily during radiation treatment. This was followed by adjuvant TMZ with a dose 150 mg/m2 daily 5 days every 28 days, as per the NCIC regimen [1]. At the time of progression, patients were assessed for second line chemotherapy, repeat surgery, and/or re-irradiation.

### Patient assessment and outcome

Patients were assessed weekly during RT with physical examination, and bloods tests including complete blood counts, serum electrolytes as well as renal and liver function. After completion of RT, patients were assessed on follow-up visit every three months with clinical evaluation and MRI. Evaluation of tumor response was determined based on imaging reports and review of the MRI T1 enhanced and/or T2 images.

The date of recurrence was determined from the time at which either progressive residual enhancement was observed or new areas of disease appeared on imaging. Progression free survival (PFS) was defined from the date of diagnosis until the documentation of disease progression clinically and/or radiologically. Overall survival (OS) was measured from date of diagnosis until date of death, or date of last clinical follow-up visit.

Pseudoprogression was defined as radiological progression within three to six months of RT completion, followed by stability or regression of the lesion without the patient receiving treatment for recurrence. Patients who had repeat surgery for presumed recurrence who did not show evidence of persistent malignancy on pathology were also deemed to have pseudoprogression. The pattern of failure was deemed to be central if in continuity with the initial tumor cavity, and distal if more than 2 cm from the cavity.

### Endpoints and statistical analysis

The primary endpoint of this retrospective study was OS and the secondary endpoint was PFS. Statistical analysis was carried out using SAS version 9.3 (SAS Institute, Cary, NC, USA). Descriptive statistics were used to summarize the study population. Univariate and multivariate Cox proportional hazard regression models were used to assess the impact of different treatment regimens on OS and PFS. The index date was date of death for analyses of OS, and date of first progression for PFS. The effect of treatment regimen on survival was quantified by means of hazard ratios (HRs) with 95% confidence intervals (95% CIs). Analyses were adjusted for important prognostic factors for survival, such as KPS (dichotomous: ≥ 70 *versus* 70), age (dichotomous: ≥ 65 *versus* <65), extent of initial surgery performed (biopsy *versus* subtotal resection or gross total resection), having any chemotherapy before the index date (dichotomous), having repeated surgery before the index date and methylation status of MGMT (methylated, unmethylated and unknown). In order to investigate artificial differences in survival caused by treatment based on age and KPS status, we conducted sensitivity analyses of multivariate models, testing for the multiplicative interaction terms between age and treatment group and between KPS status and treatment group. Analyses were independently repeated for each treatment group. We assessed the assumption of proportional hazard by examining graphs of scaled Schoenfeld residuals. Statistical significance of Kaplan-Meyer curves was assessed by the log-rank test. All analyses were two-sided with p ≤ 0.05 being considered significant.

## Results

### Patient population characteristics

A total of 276 patients with histologically-proven GBM who received adjuvant RT, with or without concomitant TMZ, were included in this population-based study. Overall median follow-up time was 13.2 months (range 1.4 to 105.7 months). Patient characteristics are summarized in Table 1. One hundred and forty-seven patients received ConvRT, 86 patients received hypofractionated RT as per the HF60, and 43 patients as per the HF40 regimen. Two hundred and two patients were found to have tumor progression on imaging. The median survival for the whole population was 13.7 months with a median PFS of 8.8 months.

**Table 1:** **Patient characteristics per treatment groups**

	**Patients (n = 276)**						
	**60 Gy in 30 Fr**		**60 Gy in 20 Fr**		**40 Gy in 15 Fr**		***P value***
	**(n = 147)**		**(n = 86)**		**(n = 43)**		
	**Number**	**%**	**Number**	**%**	**Number**	**%**	
**Age**							
<65	114	77.6	68	79.1	8	18.6	<.0001
≥65	33	22.4	18	20.9	35	81.4	
median	59		57		72		
**Sex**							
Female	55	37.4	34	39.5	20	46.5	0.562
Male	92	65.6	52	60.5	23	53.5	
**KPS**							
<70	7	4.7	10	11.6	20	46.5	<.0001
≥70	140	95.3	76	88.4	23	53.5	
**Focality**							
Multifocal	12	8.2	8	9.3	8	18.6	0.130
Unifocal	135	91.8	78	90.7	35	81.4	
**Extent of surgery**							
Biopsy	12	8.1	17	19.7	8	18.6	0.017
STR	98	66.6	45	52.3	30	63.7	
GTR	37	25.1	24	27.9	5	11.6	
**Chemotherapy**							
Yes	141	95.2	85	98.8	24	55.8	<.0001
No	4	2.7	1	1.2	16	37.2	
Unknown	2	1.3	0	0	3	6.9	
**Repeat surgery**							
Yes	49	33.3	20	23.2	2	4.6	0.001
No	98	66.7	66	76.7	41	93.4	
**MGMT**							
Methylated	60	40.8	25	29	16	37.2	0.031
Unmethylated	53	36.0	38	44.1	9	20.9	
Unknown	34	23.2	23	26.7	18	41.8	

*Abbreviations*: *Fr* Fractions, *STR* Subtotal resection, *GTR* Gross total resection, *MGMT* O6-methylguanine-DNA methyltransferase.

The similarities in patient characteristics between the ConvRT and HF60 groups are in contrast to that of patients in the HF40 group. Patients in the ConvRT and HF60 groups were more likely to have gross tumor resection (GTR), to have had repeat surgery at the time of recurrence, and to have received chemotherapy at some point during their treatment. Patients in the HF40 group were older in age, with a median age of 72, and had a more limited performance status, with close to half of these patients having a KPS of less than 70.

### Treatment regimen, OS and PFS

**Figure 1:**
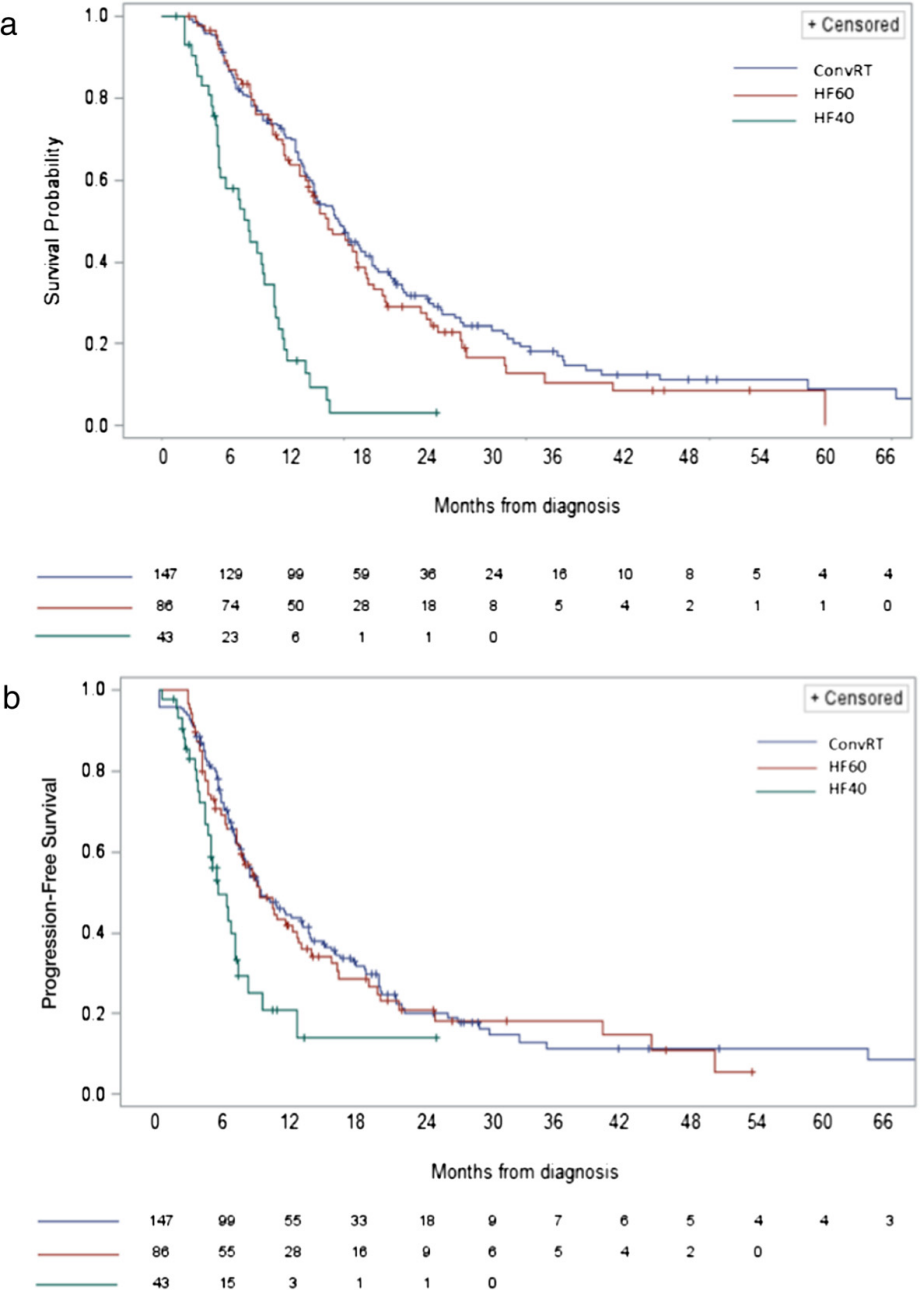
Kaplan Meier curves comparing overall survival **(a)** and progression-free survival **(b)** between treatment regimens.

Median survival was 16 months in the ConvRT group and 15 months in the HF60 group (*P* = .3487, Figure 1a). Survival in the HF40 arm was significantly lower than ConvRT with a median survival of 8 months (*P* < .00001). This difference in survival was also sustained at 1 and 2 years after diagnosis, with a 2-year OS of 23.1% and 19.7% (*P* = .347) in the ConvRT and HF60 groups, respectively. Compared to these results, the OS was extremely poor in the HF40 group, with only 2.32% of patients still alive at 2 years. Our data also showed that for patients 65 year and older, the median survival was 10.0 months in the ConvRT group, compared to 9.13 (*P* = .357) for the HF60 patients, and 7.6 months for the HF40 patients (*P* = .0049). We did not find any indication in our cohort of patients of an interaction between age and treatment status (Wald Chi-squared test = 0.0261; p = 0.8717). We detected a borderline significant interaction between KPS score and treatment regimen group (Wald Chi-squared test = 5.8949; p = 0.0525).

These trends were also observed in terms of PFS (Figure 1b). Median PFS was close to 9 months in both the ConvRT and the HF60 groups, compared to 5.4 months in the HF40 groups (*P* = .0002). The PFS at 1 year was 37.4% in the ConvRT group, 31.4% in the HF60 group (*P* = .6894) and 7.0% in the HF40 group (*P* = .0007).

### Prognostic factors associated with survival

Multivariate analysis showed that treatment regimen (ConvRT and HF60), methylation status, use of chemotherapy, extent of resection and repeat surgery at the time of recurrence were the most significant independent prognostic factors for survival (Table 2). There was no significant difference in treatment outcome when HF60 was compared to ConvRT (HR: 1.27, 95% CI, 0.93-1.74). This finding is in contrast to the HF40 treatment group, which showed significantly worse outcome (HR 2.22; 95% CI, 1.30 to 3.80).

**Table 2:** **Univariate and multivariate analysis of variables affecting overall survival**

	**Univariate analysis**			**Multivariate analysis**		
	**HR**	**95% CI**	***P value***	**HR**	**95% CI**	***P value***
**Age**						
<65	1			1		
≥65	2.14 (1.60-2.86)		<.0001	1.25 (0.86-1.82)		0.235
**KPS**						
<70	1			1		
≥70	0.51 (0.35-0.74)		0.0004	0.67 (0.43-1.05)		0.078
**Extent of surgery**						
Biopsy	1			1		
STR	0.54 (0.38-0.79)		0.0013	0.62 (0.39-0.99)		0.048
GTR	0.36 (0.23-0.56)		<.0001	0.39 (0.22-0.67)		0.001
**Treatment**						
60 Gy/30 fr	1			1		
60 Gy/20 fr	1.15 (0.86-1.56)		0.3475	1.27 (0.93-1.74)		0.126
40 Gy/15 fr	4.07 (2.75-6.04)		<.0001	2.22 (1.30-3.80)		0.004
**Chemotherapy**						
No	1			1		
Yes	0.20 (0.12-0.34)		<.0001	0.52 (0.28-0.99)		0.045
**Repeat surgery**						
No	1			1		
Yes	0.51 (0.38-0.70)		<.0001	0.62 (0.44-0.86)		0.005
**MGMT**						
Unmethylated	1			1		
Methylated	0.53 (0.39-0.74)		0.0001	0.46 (0.33-0.64)		<.0001

*Abbreviations*: *HR* Hazard ratio, *CI* Confident interval, *FR* Fractions, *STR* Subtotal resection, *GTR* Gross total resection, *MGMT* O6-methylguanine-DNA methyltransferase.

Patients with MGMT promoter methylation showed significantly better survival compared to unmethylated patients (HR 0.46 95% CI, 0.33 to 0.64; *P* < .0001). This was seen in both the HF60 and ConvRT treatment regimens. There were no independent prognostic factor identified in the 65 years-old and older subgroup of patients. It must be noted that sample size for this population was small (n = 86) and that MGMT status could not be determined in half of these patients due to limited tissue sampling.

Extent of resection was also found to significantly affect outcome. At the time of initial surgery, both STR (HR 0.62; CI, 0.39-0.99; *P* = .048) and GTR (HR 0.39; CI, 0.22 to 0.67; *P* = .001) were found to be superior to biopsy alone. The effect of repeat debulking surgery at the time of recurrence was also found to correlate with survival (HR 0.62; CI, 0.44 to 0.86; *P* = .005). Upon looking at factors affecting individual treatment regimens, we found that in both the ConvRT and HF60 groups, methylation status, and extent of resection were the most significant prognostic factors affecting OS (Table 3). In addition, in the HF60 group, age, use of chemotherapy and repeat surgery also correlated with better outcome. In comparison to ConvRT, only performance status significantly affected survival in the HF40 group.

**Table 3:** **Multivariate analysis of prognostic factor for OS in each treatment group**

	**Treatment groups**								
	**60 Gy in 30 fr**			**60 Gy in 20 fr**			**40 Gy in 15 fr**		
	**HR**	**95% CI**	***P value***	**HR**	**95% CI**	***P value***	**HR**	**95% CI**	***P value***
**Age**									
(≥65 vs. <65)	1.58 (0.94-2.65)		0.085	2.05 (1.04-4.06)		0.039	0.69 (0.18-2.60)		0.58
**KPS**									
(≥70 vs <70)	1.52 (0.53-4.38)		0.443	0.63 (0.27-1.47)		0.285	0.27 (0.11-0.66)		0.004
**Extent of surgery**									
(STR vs. biopsy)	0.47 (0.19-1.17)		0.103	0.62 (0.30-1.25)		0.179	1.29 (0.33-0.99)		0.713
(GTR vs. biopsy)	0.33 (0.12-0.90)		0.031	0.29 (0.13-0.66)		0.003	0.20 (0.04-1.15)		0.071
**Chemotherapy**									
(Yes vs. no)	0.34 (0.10-1.12)		0.075	0.11 (0.01-0.97)		0.047	0.62 (0.24-1.62)		0.330
**Repeat surgery**									
(Yes vs. no)	0.73 (0.48-1.12)		0.149	0.35 (0.18-0.69)		0.002	0.18 (0.02-1.89)		0.154
**MGMT**									
(Methylated vs. unmethylated)	0.46 (0.29-0.72)		0.001	0.37 (0.20-0.68)		0.002	0.57 (0.18-1.78)		0.329

*Abbreviations*: *FR* Fractions, *STR* Subtotal resection, *GTR* Gross total resection, *MGMT* O6-methylguanine-DNA methyltransferase.

Pseudoprogression was found to develop at a median time of 3.8 months in 10.8% of patients. Patients who developed pseudoprogression had a median survival of 25.16 months with a 2- year OS of 46.6%, vs. a median survival of 13.4 months and a 2-year OS of 16.6% for those who did not (*P* = .002). Pseudoprogression did not show an association with either the MGMT methylation status of the tumor (*P* = .4506) or the RT regimen received (*P* = .70).

### Impact of methylation of MGMT on survival and pattern of recurrence

**Figure 2:**
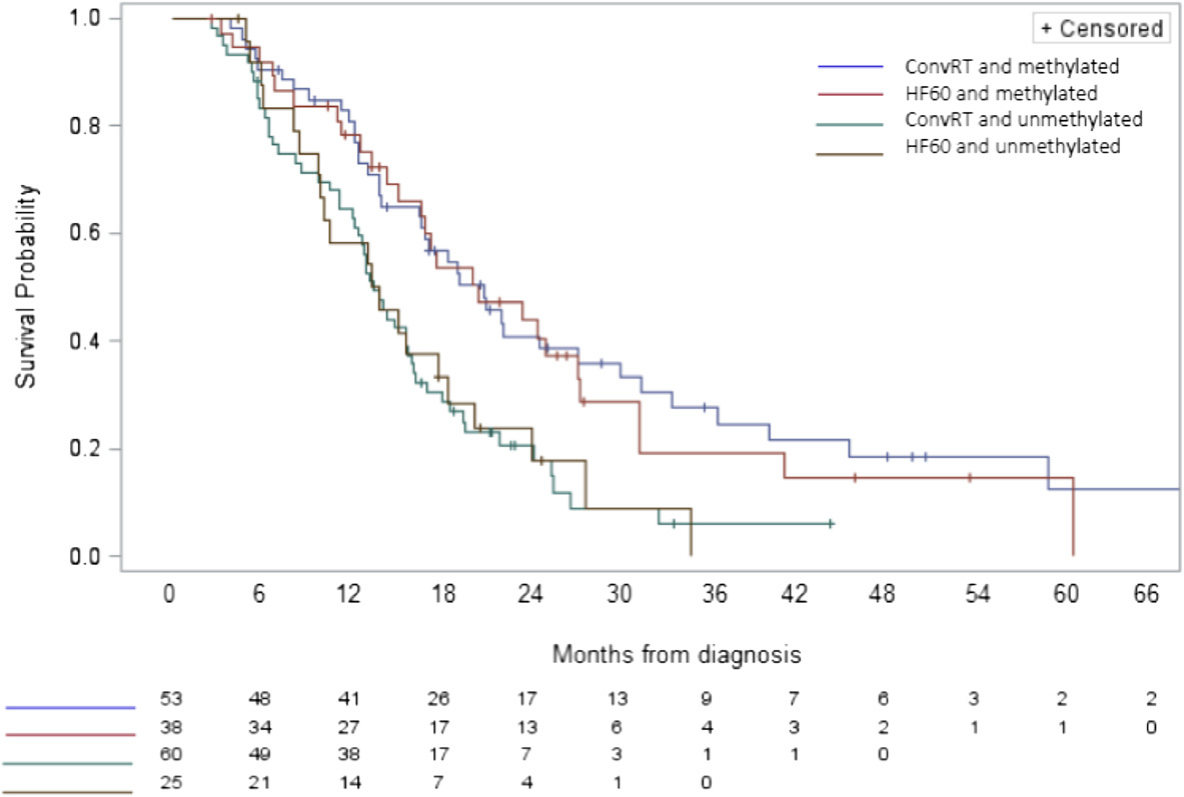
Kaplan Meier cure for overall survival showing 2 fractionation regimens based on the methylation status of the MGMT promoter.

Methylation of MGMT promoter was found as an independent prognostic factor for OS. On Kaplan-Meier analysis, median survival in the ConvRT group of patients with MGMT promoter methylation was 21 months, vs. 13.4 months for unmethylated tumors (*P* = .001; Figure 2). Similar results were seen in the HF60 group, with MS of 20.6 months for methylated tumors, vs. 13.6 months for unmethylated tumors (*P* = .0325). In the HF40 group, patients with methylated tumour had a median survival of 10.2 months, compared to 7.9 months for unmethylated tumours. The outcome of patients with methylated (*P* = .674) or unmethylated MGMT (*P* = .891) was not affected by the RT regimen being used.

The pattern of recurrence was evaluated and correlated with the MGMT methylation status. We found that distal recurrences were more frequently associated with MGMT methylated tumors (*P* = .020). We have also identified a trend for central recurrence to be more often linked to unmethylated tumors (*P* = .070). However, both methylated and unmethylated tumors were more likely to recur centrally than to recur distally (*P* < .0001).

## Discussion

The combination of RT and TMZ followed by adjuvant TMZ has allowed to improve outcome for GBM patients [1]. Despite the modest improvement in survival, this regimen involves a prolonged RT course for patients with reduced life expectancy. For that reason, defining the optimal RT regimen is necessary. In this population-based study, we have compared two GBM treatment regimens to the ConvRT schedule. This is the first analysis showing that GBM treatment with hypofractionated RT (HF60) is associated with comparable outcome to conventional Stupp regimen [1].

Our findings corroborate the impact on survival of some prognostic factors that have been previously reported [18-20]. Here, the MGMT methylation status was found to be a strong independent prognostic factor for survival regardless the type of regimen used (HF60 and ConvRT). Another factor strongly associated with improved outcome is the extent of the initial tumor resection [21-23]. We observed that maximal safe resection correlates with improved outcome when compared to biopsy (HR 0.39) or subtotal resection (HR 0.62). In addition, the repeat surgery at the time of progression improves patient outcome on multivariate analysis (HR 0.62). Whether this is due to patient selection bias due to re-operating mostly fit patients with more accessible tumors should be considered.

Our own experience regarding the use of hypofractionation dates back to 1994. A phase I-II trial was conducted by Shenouda et al. using a concomitant boost technique [24]. The treatment was equivalent to that of published series with respect to toxicity and outcome at that time. Sultanem et al. reported on 25 patients who received 60 Gy in 20 fractions to the gross tumor volume, with concurrent administration of 40 Gy in 20 fractions to the PTV [9]. The median survival was 9.5 months, with a 1-year survival rate of 40%, and median PFS of 5.2 months. Panet-Raymond et al. further reported on this RT regimen, with the inclusion of concomitant and adjuvant TMZ [10]. These results were comparable to the survival data from the NCIC/EORTC study and from the present study, supporting the use of hypofractionation as a valid RT regimen.

The optimal management for GBM in elderly patients remains poorly defined due to the heterogeneity of this population in terms of performance status, co-morbidity, and treatment options [25]. Roa et al. reported that adjuvant hypofractionated RT (dose of 40 Gy in 15 fractions) without TMZ was associated with the same survival when compared to conventionally fractionated RT (60 Gy in 30 fractions) in elderly patients [26]. In contrast, the NORDIC randomized trial showed that hypofractionated (34 Gy in 10 fractions) was superior to conventionally fractionated RT in that group of patients [8]. Despite these controversies, there is growing evidence for the use of hypofractionation in elderly GBM patients to shorten treatment duration and overcome radiation resistance [8,26-28]. In our practice, the ConvRT and HF60 regimens are generally offered to patients with good performance status who underwent maximum safe resection. This approach yields good results, with better OS compared to treatment with the HF40 regimen, including for patients 65 years of age or older. Admittedly, the HF40 regimen is typically offered to patient with poor KPS and limited tumor resection, factors that could predict worse survival. However, our multivariate analysis which controlled for these factors showed that patients receiving the HF40 regimen are twice more likely to die compared to ConvRT. These findings support that a more radical approach can be considered for selected elderly GBM patients with good performance status and limited comorbidities [25,29].

The outcome of patients receiving ConvRT can be compromised by tumor repopulation [30]. It has been suggested that hypofractionation can be more effective from a radiobiological standpoint to overcome the radioresistance of GBM cells. It has the dual effects of increasing cell death from a higher dose per fraction, and reducing the effect of tumor repopulation [6]. The biological effective dose (BED) is used to clinically compare different fractionation regimens in terms of effectiveness. Assuming an α/β of 3 for late responding neural tissue and α/β of 10 for tumor cells, the BED_3_ for the ConvRT regimen would be 100 Gy with a BED_10_ of 72 Gy, for GBM tumor cells. For the HF60 regimen, the BED_3_ would be 120 Gy and the BED_10_ would be 78 Gy. The BED for the HF40 regimen is much lower, with a BED_3_ of 75 Gy and a BED_10_ of 50.5 Gy. While the HF60 shows a higher BED for tumor cells, our own comparison did not demonstrate the superiority of the hypofractionated RT approach compared to ConvRT in terms of OS. More investigation is needed to better evaluate which fractionation regimen and dose, delivered in the presence of concomitant TMZ, could potentially overcome the inherent radioresistance of GBM cells [31].

We acknowledge the limitations of the present study, including the lack of data regarding long-term treatment-related toxicity, as well as the heterogeneity of the treatment groups, which reflects the population-based nature of our study. Based on our experience using IMRT-based HF60 over the last 10 years, cases of radiation-induced toxicities have been rare, with most patients tolerating hypofractionated RT in combination with TMZ without complications. The use of advanced delivery technologies, such as IMRT and image-guided RT, have allowed for more conformal treatment delivery, thereby decreasing the risk of serious long-term toxicities. Furthermore, the potential benefits in terms of reduction of the financial burden on the health care system and improved quality of life for patients from a shortened course of RT should not be neglected.

In conclusion, moderate hypofractionated RT (HF60) is associated with comparable outcome to conventional RT regimen for newly diagnosed GBM patients. Our study also confirms the prognostic value of several factors, including MGMT promoter methylation and re-operation at the time of recurrence. However, the optimal treatment for elderly population remains elusive and more research is necessary to better adapt treatment for these patients. Identification of biomarkers of responsiveness to hypofractionation is necessary to better tailor treatment in newly diagnosed GBM patients.

## References

[1] Stupp R, Mason WP, van den Bent MJ, Weller M, Fisher B, Taphoorn MJ (2005). Radiotherapy plus concomitant and adjuvant temozolomide for glioblastoma. N Engl J Med.

[2] Marko NF, Weil RJ, Schroeder JL, Lang FF, Suki D, Sawaya RE (2014). Extent of resection of glioblastoma revisited: personalized survival modeling facilitates more accurate survival prediction and supports a maximum-safe-resection approach to surgery. J Clin Oncol.

[3] Wen PY, Macdonald DR, Reardon DA, Cloughesy TF, Sorensen AG, Galanis E (2010). Updated response assessment criteria for high-grade gliomas: response assessment in neuro-oncology working group. J Clin Oncol.

[4] Stupp R, Hegi ME, Mason WP, van den Bent MJ, Taphoorn MJ, Janzer RC (2009). Effects of radiotherapy with concomitant and adjuvant temozolomide versus radiotherapy alone on survival in glioblastoma in a randomised phase III study: 5-year analysis of the EORTC-NCIC trial. Lancet Oncol.

[5] Laperriere N, Zuraw L, Cairncross G, Cancer Care Ontario Practice Guidelines Initiative Neuro-Oncology Disease Site Group (2002). Radiotherapy for newly diagnosed malignant glioma in adults: a systematic review. J European Soc Ther Radiol Oncol.

[6] Hingorani M, Colley WP, Dixit S, Beavis AM (2012). Hypofractionated radiotherapy for glioblastoma: strategy for poor-risk patients or hope for the future?. Br J Radiol.

[7] Mirimanoff RO, Gorlia T, Mason W, Van den Bent MJ, Kortmann RD, Fisher B (2006). Radiotherapy and temozolomide for newly diagnosed glioblastoma: recursive partitioning analysis of the EORTC 26981/22981-NCIC CE3 phase III randomized trial. J Clin.

[8] Malmstrom A, Gronberg BH, Marosi C, Stupp R, Frappaz D, Schultz H (2012). Temozolomide versus standard 6-week radiotherapy versus hypofractionated radiotherapy in patients older than 60 years with glioblastoma: the Nordic randomised, phase 3 trial. Lancet Oncol.

[9] Sultanem K, Patrocinio H, Lambert C, Corns R, Leblanc R, Parker W (2004). The use of hypofractionated intensity-modulated irradiation in the treatment of glioblastoma multiforme: preliminary results of a prospective trial. Int J Radiat Oncol Biol Phys.

[10] Panet-Raymond V, Souhami L, Roberge D, Kavan P, Shakibnia L, Muanza T (2009). Accelerated hypofractionated intensity-modulated radiotherapy with concurrent and adjuvant temozolomide for patients with glioblastoma multiforme: a safety and efficacy analysis. Int J Radiat Oncol Biol Phys.

[11] Ciammella P, Galeandro M, D’Abbiero N, Podgornii A, Pisanello A, Botti A (2013). Hypo-fractionated IMRT for patients with newly diagnosed glioblastoma multiforme: A 6 year single institutional experience. Clin Neurol Neurosurg.

[12] Reddy K, Damek D, Gaspar LE, Ney D, Waziri A, Lillehei K (2012). Phase II trial of hypofractionated IMRT with temozolomide for patients with newly diagnosed glioblastoma multiforme. Int J Radiat Oncol Biol Phys.

[13] Floyd NS, Woo SY, Teh BS, Prado C, Mai WY, Trask T (2004). Hypofractionated intensity-modulated radiotherapy for primary glioblastoma multiforme. Int J Radiat Oncol Biol Phys.

[14] Jastaniyah N, Murtha A, Pervez N, Le D, Roa W, Patel S (2013). Phase I study of hypofractionated intensity modulated radiation therapy with concurrent and adjuvant temozolomide in patients with glioblastoma multiforme. Radiat Oncol.

[15] Iuchi T, Hatano K, Kodama T, Sakaida T, Yokoi S, Kawasaki K (2014). Phase 2 trial of hypofractionated high-dose intensity modulated radiation therapy with concurrent and adjuvant temozolomide for newly diagnosed glioblastoma. Int J Radiat Oncol Biol Phys.

[16] Chen C, Damek D, Gaspar LE, Waziri A, Lillehei K, Kleinschmidt-DeMasters BK (2011). Phase I trial of hypofractionated intensity-modulated radiotherapy with temozolomide chemotherapy for patients with newly diagnosed glioblastoma multiforme. Int J Radiat Oncol Biol Phys.

[17] Network NCc. NCCN Clinical Practise Guidelines in Oncology: Central nervous system. 2014. http://www.nccn.org.

[18] Gorlia T, van den Bent MJ, Hegi ME, Mirimanoff RO, Weller M, Cairncross JG (2008). Nomograms for predicting survival of patients with newly diagnosed glioblastoma: prognostic factor analysis of EORTC and NCIC trial 26981-22981/CE.3. Lancet Oncol.

[19] Lamborn KR, Chang SM, Prados MD (2004). Prognostic factors for survival of patients with glioblastoma: recursive partitioning analysis. Neuro Oncol.

[20] Hegi ME, Liu L, Herman JG, Stupp R, Wick W, Weller M (2008). Correlation of O6-methylguanine methyltransferase (MGMT) promoter methylation with clinical outcomes in glioblastoma and clinical strategies to modulate MGMT activity. J Clin Oncol.

[21] Stummer W, Meinel T, Ewelt C, Martus P, Jakobs O, Felsberg J (2012). Prospective cohort study of radiotherapy with concomitant and adjuvant temozolomide chemotherapy for glioblastoma patients with no or minimal residual enhancing tumor load after surgery. J Neurooncol.

[22] Kreth FW, Thon N, Simon M, Westphal M, Schackert G, Nikkhah G (2013). Gross total but not incomplete resection of glioblastoma prolongs survival in the era of radiochemotherapy. Annals Oncol J European Soc Med Oncol.

[23] De Bonis P, Anile C, Pompucci A, Fiorentino A, Balducci M, Chiesa S (2013). The influence of surgery on recurrence pattern of glioblastoma. Clin Neurol Neurosurg.

[24] Shenouda G, Souhami L, Freeman CR, Hazel J, Lehnert S, Joseph L (1991). Accelerated fractionation for high-grade cerebral astrocytomas. Preliminary treatment results. Cancer.

[25] Fiorentino A, Balducci M, De Bonis P, Chiesa S, De Filippo L, Mangiola A (2015). Can elderly patients with newly diagnosed glioblastoma be enrolled in radiochemotherapy trials?. Am J Clin Oncol.

[26] Roa W, Brasher PM, Bauman G, Anthes M, Bruera E, Chan A (2004). Abbreviated course of radiation therapy in older patients with glioblastoma multiforme: a prospective randomized clinical trial. J Clin Oncol.

[27] Wick W, Platten M, Meisner C, Felsberg J, Tabatabai G, Simon M (2012). Temozolomide chemotherapy alone versus radiotherapy alone for malignant astrocytoma in the elderly: the NOA-08 randomised, phase 3 trial. Lancet Oncol.

[28] Minniti G, Lanzetta G, Scaringi C, Caporello P, Salvati M, Arcella A (2012). Phase II study of short-course radiotherapy plus concomitant and adjuvant temozolomide in elderly patients with glioblastoma. Int J Radiat Oncol Biol Phys.

[29] Bauchet L, Zouaoui S, Darlix A, Menjot de Champfleur N, Ferreira E, Fabbro M, et al. Assessment and treatment relevance in elderly glioblastoma patients. Neuro Oncol. 2014. doi:10.1093/neuonc/nou063. 10.1093/neuonc/nou063 PMC4201066 24792440

[30] Budach W, Gioioso D, Taghian A, Stuschke M, Suit HD (1997). Repopulation capacity during fractionated irradiation of squamous cell carcinomas and glioblastomas in vitro. Int J Radiat Oncol Biol Phys.

[31] Pedicini P, Fiorentino A, Simeon V, Tini P, Chiumento C, Pirtoli L (2014). Clinical radiobiology of glioblastoma multiforme: estimation of tumor control probability from various radiotherapy fractionation schemes. Strahlenther Onkol.

